# SSB1/SSB2 Proteins Safeguard B Cell Development by Protecting the Genomes of B Cell Precursors

**DOI:** 10.4049/jimmunol.1801618

**Published:** 2019-05-13

**Authors:** Matthias Pfeifer, Reto Brem, Timothy P. Lippert, Bryant Boulianne, Howin Ng Ho, Mark E. Robinson, Justin Stebbing, Niklas Feldhahn

**Affiliations:** * Centre for Hematology, Department of Medicine, Imperial College London, W12 0NN London, United Kingdom; and; † Division of Cancer, Department of Surgery and Cancer, Imperial College London, W12 0NN London, United Kingdom

## Abstract

Induction of programmed DNA damage and its recognition and repair are fundamental for B cell development. The ssDNA-binding protein SSB1 has been described in human cells as essential for the recognition and repair of DNA damage. To study its relevance for B cells, we recently developed *Ssb1*^−/−^ and conditional *Ssb1*^−/−^ mice. Although SSB1 loss did not affect B cell development, *Ssb1*^−/−^ cells exhibited compensatory expression of its homolog SSB2. We have now generated *Ssb2*^−/−^ mice and show in this study that SSB2 is also dispensable for B cell development and DNA damage response activation. In contrast to the single loss of *Ssb1* or *Ssb2*, however, combined SSB1/2 deficiency caused a defect in early B cell development. We relate this to the sensitivity of B cell precursors as mature B cells largely tolerated their loss. Toxicity of combined genetic SSB1/2 loss can be rescued by ectopic expression of either SSB1 or SSB2, mimicked by expression of SSB1 ssDNA-binding mutants, and attenuated by BCL2-mediated suppression of apoptosis. SSB1/2 loss in B cell precursors further caused increased exposure of ssDNA associated with disruption of genome fragile sites, inefficient cell cycle progression, and increased DNA damage if apoptosis is suppressed. As such, our results establish SSB1/2 as safeguards of B cell development and unveil their differential requirement in immature and mature B lymphocytes.

## Key Points

- Combined deficiency of *Ssb1* and *Ssb2* abrogates early B cell development.
- SSB1/2 depletion causes defects in proliferation, genome fragility, and apoptosis.
- Mature B cells largely tolerate SSB1/2 loss.

## Introduction

Deoxyribonucleic acid is the carrier of genetic information but is inherently unstable. Although it is constantly threatened by exogenous or endogenous damage, a variety of DNA damage repair mechanisms ensure the maintenance of its integrity (1). B and T lymphocytes, in addition, induce programmed DNA damage at their Ig and TCR loci as part of their normal differentiation program. The underlying processes, V(D)J recombination during early B and T cell differentiation and Ig class-switch recombination (CSR) in mature B cells, rely on the repair of induced DNA double-strand breaks (DSB) by the nonhomologous end-joining (NHEJ) repair pathway. Accordingly, defects in NHEJ components typically impair B cell and T cell development (2, 3). In contrast, DNA damage response (DDR) and repair proteins not related to NHEJ are usually dispensable for B and T cell development. However, highlighting their crucial role in safeguarding the lymphocyte genome, mice deficient for DDR genes are prone to develop lymphoid malignancies (4).

Numerous studies over the last decade have characterized human ssDNA-binding protein 1 (hSSB1) as an integral component of DNA damage recognition and repair pathways. hSSB1, also known as oligonucleotide/oligosaccharide-binding–fold domain-containing protein 2B (OBFC2B) or nucleic acid-binding protein 2 (NABP2), is part of a heterotrimeric protein complex with INTS3 and C9ORF80 (5–8) and mediates recruitment of this complex to sites of DNA damage through the ability to bind ssDNA (9, 10). This complex can be similarly formed with its homolog hSSB2 (OBFC2A/NABP1), which is functionally related to hSSB1 but less well studied. In human cells, SSB1 has been shown to be crucial for the activation of the DDR, including DNA damage-induced phosphorylation of ATM, P53, CHK1, and CHK2 (6, 7, 9), and the activation of the G_1_/S and G_2_/M cell cycle checkpoints in response to DNA damage (5, 9). In mice, the requirement of SSB1 for DDR activation is less apparent and varies between individual mouse models (10–12). hSSB1 has been further described to mediate recruitment of the MRN complex to DNA DSB (5, 13, 14), the repair of DNA damage by homologous recombination (5, 9), and the repair of stalled replication forks and oxidative stress–induced DNA damage (15, 16). In addition, two studies suggested human and mouse SSB1 to be potentially important for NHEJ (10, 17). Mouse models by others and us further revealed that murine SSB1 is crucial for bone formation during normal embryogenesis. Depletion led to perinatal lethality (12), whereas induced loss in mature mice increased the prevalence of lymphoid malignancies in mid or late age, depending on the mouse model (10, 11). Murine SSB1 was also shown to protect telomeres in cells expressing a dominant-negative isoform of the Shelterin complex component TPP1 (10).

We initially generated our *Ssb1*^−/−^ mouse model to study its function in B cells. Although we did not observe any B cell–specific phenotypic abnormalities caused by SSB1 loss, B cells, as well as many other cellular compartments, reacted to SSB1 protein loss with increased expression of its homolog SSB2 (12), which likely masked the phenotype of SSB1 loss. To better study its function in B cells and extend our previous work, we proceeded to generate *Ssb2* knockout (KO) and *Ssb1/2* conditional double KO (cDKO) mice. In this study, we show that SSB1/2 proteins are essential for normal B cell development, uncover their differential requirement during early and late stages of B cell development, and link SSB1/2 function to the stabilization of chromosomal fragile sites.

## Materials and Methods

### Mice

Conditional *Ssb1* KO mice and transgenic *Cd19Cre* and *Cre-ERT2* strains are described (see 12, 18, 19). *Ssb2*^−/−^ mice were derived at The Rockefeller University, New York, NY, from targeted embryonic stem (ES) cells generated at the Helmholtz Zentrum München (Munich, Germany) as part of the European Conditional Mouse Mutagenesis Program. The ES clone used to generate the line was EUCE0058b05 and was generated using gene trap (GT) technology (20). Integration of the GT cassette at the *Ssb2* locus was confirmed using Sanger sequencing. Mice were bred within the C57BL/6 background. For *Cd19*Cre cDKO experiments, 6- to 8-wk-old mice were analyzed, and for *ERT2-Cre* cDKO experiments, 6- to 12-wk-old mice were used for purification of bone marrow (BM) and spleen B cells. All experiments were in agreement with protocols approved by the Rockefeller University and National Institutes of Health Institutional Animal Care and Use Committee for experiments performed in the United States and with the Animals Scientific Procedures Act 1986 guidelines, regulations, and protocols approved by the Home Office for experiments performed in the United Kingdom.

### Cell culture

For ex vivo culture of primary B cell precursors, BM was isolated from femur and tibia of mice and depleted from erythrocytes using ACK lysis buffer (Life Technologies). Primary B cell precursors were cultured in the presence of 10 ng/ml recombinant mouse IL-7 (Peprotech) as previously described (21). Mature B cells were isolated from spleen and depleted from CD43^+^ cells by magnetic cell sorting and anti-mouse CD43 microbeads (Miltenyi Biotech). Mature B cells were cultured ex vivo in the presence of 25 μg/ml LPS (Sigma-Aldrich) and 5 ng/ml mouse rIL-4 (Sigma-Aldricha) as previously described (22). Irradiation (IR) of cells was performed at time points, dosages, and recovery times as indicated in the figures. Murine B cell precursor cell lines were generated using IL-7–cultured primary B cell precursors from *Cre-ERT2*^+^ cDKO and *Ssb2-*deficient control (Ctrl) mice and c-MYC encoding retrovirus, as previously described (21). Successfully transformed cell lines represent cells at >50 d posttransduction (>80% viability) and were continuously cultured in the presence of IL-7. B cell precursor cell lines were further transduced using SSB1, SSB2, BCL2, sRPA, or empty vector (EV) encoding retrovirus; Puromycin selected; and used for experiments as indicated. For genetic SSB1/2 ablation, *Cre-ERT2*^+^ Ctrl and cDKO cells were equally treated with 0.25 or 0.125 μM OHT (Sigma-Aldrich) and assayed at time points indicated in the figure legends. Viability was monitored using trypan blue exclusion or flow cytometry, as indicated in each figure. All cultured cells were maintained at 37°C in 5% CO_2._ For IL-7 withdrawal experiments, 20 ng/ml murine BAFF (BioLegend) and 25 μg/ml LPS were used, and residual IgM^+^ cells (∼1–2%) were depleted before the start of each experiment using magnetic cell sorting and anti-mouse IgM microbeads (Miltenyi Biotech).

### Viral vectors and transduction

Coding sequences of murine *Ssb1* and *Ssb2* were amplified using Q5 High-Fidelity Polymerase (NEB) and cDNA of LPS/IL-4–treated murine splenic B cells using the following primers: 5′-GGGATCCGCCGCCATGACGACCGAGACCTTCG-3′ and 5′-GCTCGAGCTACTTGTCGTCGTCGTCCTTGTAGTCGCCGCCTGAGCCTCTCTTGCTGCTTCTTCGG-3′ (*Ssb1*); 5′-GGGATCCGCCGCCATGCACGGGGTCAACGACC-3′ and 5′-GCTCGAGCTACTTGTCGTCGTCGTCCTTGTAGTCGCCGCCTGAGCCTCTTTTAAAGGCTCTCCTCGGATC-3′ (*Ssb2*).

Amplified coding sequences were then cloned into the expression vector pMX (coexpressing GFP or dsRED, respectively) using BamHI and XhoI for both *Ssb1* and *Ssb2*. ssDNA-binding mutants of SSB1 were generated by site-directed mutagenesis using the QuikChange Site-Directed Mutagenesis Kit (Agilent) and the following primers: 5′-CAACATCTCAGTAGCGGACGATGTGGGC-3′ and 5′-GCCCACATCGTCCGCTACTGAGATGTTG-3′ (W55A); 5′-CTCACCAAAGGGGCTGCTTCAGTGTTCAAAGG-3′ and 5′-CCTTTGAACACTGAAGCAGCCCCTTTGGTGAG-3′ (Y74A).

The expression vector pBABE was used to express *Bcl2* cDNA (Addgene). The sRPA plasmid was a kind gift of L. Toledo. sRPA was further subcloned into pMX–GFP vectors using Q5 High-Fidelity Polymerase (NEB) and the following primers: 5′-GATCGAATTCGCCGCCATGGTGGACATGATGGACTTGC-3′ and 5′-GATCGAATTCTTATTCTGCATCTGTGGATTTAAAATGGTCA-3′ (sRPA).

 Virus production of all vectors was achieved through transfection of BOSC293T, as previously described (22), using Xtremegene9 (ROCHE) and pCL-ECO (Addgene) as helper plasmid. Viral transductions were done by two consecutive rounds of spinoculation, as described (21). For experiments indicated, selection of transduced cells was maintained using 1 μg/ml Puromycin (Sigma-Aldrich).

### Abs and dyes

Western blot and flow cytometry were performed as described (21). For detection of specific proteins by Western blot, the following Abs were used: anti-phospho-histone H2A.X (serine 139) (γH2AX) clone JBW301 (Millipore), anti-phospho-P53 (serine 15) (Cell Signaling), anti-phospho-CHK1 (serine 317) (R&D), anti-β-ACTIN (clone W16197A; BioLegend), anti-α-TUBULIN (Abcam), anti-LAMIN B1 (Abcam), anti-hSSB1/OBFC2B (SSB1) (Bethyl Laboratories), anti-OBFC2A (SSB2) (Proteintech Group), anti-phospho-KAP1 (serine 824) (Bethyl Laboratories), anti-FLAG-HRP (Sigma-Aldrich), anti-RPA1 (Bethyl Laboratories), anti-RPA2 (clone NA19L; Calbiochem), anti-RPA3 (Abcam), anti-Vinculin (Cell Signaling), and anti-BCL2 (Cell Signaling). For cell surface staining of single-cell suspensions and consecutive analysis by flow cytometry, the following anti-mouse Abs were used: anti-CD16/32 (Fc Block, clone 2.4G2) (BD Biosciences), anti-CD19-APC (eBio103), anti-B220-PerCPCy5.5 (RA3-6B2), anti-IgM-APCe780 (II/41), anti-IgK-PE-Cy7 (187.1) (BD Biosciences), anti-CD43-PE (eBioR2/60), anti-CD24-FITC (30-F1), anti-IgD-e450 (11-26c), anti-CD21-FITC (eBio4E3), anti-CD23-PE (B3B4), anti-CD3-APC (17A2), anti-CD8-FITC (53-6.7), anti-CD4-PE (RM4-4), and anti-TCR-APC-Cy7 (H57-597). For analysis of Ig CSR, an anti-IgG1-APC Ab (clone A85.1; BD Biosciences) was used. For analysis of mitosis-specific markers, cells were stained using the anti-phosphorylated-histone 3 Ab (serine 10, clone D2C8), according to the manufacturer’s protocol (Cell Signaling), and analyzed using a BD LSR Fortessa flow cytometer (BD Biosciences). For native BrdU staining, anti-BrdU (clone MoBU-1; BioLegend) and Alexa Fluor 488–conjugated anti-mouse IgG Abs (clone A-11001; Invitrogen) were used. For Annexin V staining, the Annexin V and allophycocyanin conjugate (Invitrogen) was used in combination with reagents and protocols provided by the Annexin V Apoptosis Detection Kit (BD Biosciences), and cells were analyzed using a BD FACSCalibur or BD LSRFortessa. For all flow cytometric analyses, a minimum of 20,000 cells were analyzed each sample. Except for BrdU and Annexin V analyses, all flow cytometric analyses cells were gated for live cells using forward light scatter/side light scatter or the cell viability dyes 7-AAD (BD Biosciences) or Aqua (Life Technologies), as indicated in the figure legends.

### CFSE analysis

Cells were stained with 2.5 μM CFSE (BioLegend) and processed according to manufacturer’s instructions. Cells were then analyzed at indicated time points using a FACS Calibur (BD Biosciences).

### BrdU analyses

To monitor cell cycle progression, cells were treated for 48 h with OHT, washed with PBS, and reincubated in normal medium. Seventy-two hours later, cells were BrdU pulse labeled for 30 min; processed using the BD Pharmingen BrdU-APC kit, according to manufacturer’s protocol (including DNase treatment); and analyzed using a BD LSR Fortessa flow cytometer (BD Biosciences). For native BrdU staining, cells were OHT treated for 48 h, allowed to recover for 24 h, and then BrdU labeled for 18 h. Cytoplasm was then pre-extracted by incubation with ice-cold BrdU permeabilization buffer (10 mM PIPES [pH 7], 100 mM NaCl, 300 mM sucrose, 3 mM MgCl_2_, 0.5% Triton X-100) on ice for 15 min. Cells were immediately spun onto frosted slides using a Shandon Cytospin2 and fixed with 2% methanol-free formaldehyde in PBS for 15 min. Formaldehyde was removed by several washes in PBS, and slides were stored in PBS/0.05% sodium azide at 4°C. Slides were blocked in Ab dilution buffer (10% goat serum, 0.1% Triton X-100, 0.1% saponin in PBS) for 30 min at room temperature (RT) and stained with anti-BrdU Ab (BioLegend) (1:500 in blocking buffer) for 1 h at RT followed by Alexa Fluor 488–coupled goat anti-mouse Ab (Invitrogen) (1:500) staining for 1 h at RT. Slides were washed in PBS, briefly dipped in water, and a glass cover slip was mounted using ProLong Gold with DAPI (ThermoFisher).

### ssDNA-binding assay

ssDNA binding of SSB1 variants was analyzed, as previously described (10). Briefly, 293T cells were transfected with retroviral expression vectors encoding FLAG-tagged SSB1 variants. Three days after, transfection cells were lysed and whole-cell lysates were incubated with Oligo-dT_40_ coupled to MyOne Streptavidin T1 beads (Invitrogen). FLAG-tagged proteins bound to Oligo-dT_40_–coupled beads were separated by magnetic separation and analyzed by Western blot.

### DNA fluorescence in situ hybridization

For telomere fluorescence in situ hybridization (FISH), cells were incubated in prewarmed 0.075 M KCl at 37°C for 15 min and fixed in ice-cold methanol/acetic acid (3:1). Metaphase spreads were obtained by dropping fixed cells on frosted slides submerged in 40–45% acetic acid and air drying. Slides were air dried and rehydrated, fixed in 4% formaldehyde/PBS for 4 min, and treated with 1 mg/ml pepsin in 10 mM glycine (pH 2) for 15 min at 37°C. This was followed by washing in PBS, refixation in 4% formaldehyde in PBS for 2 min, and dehydration with an ethanol series (70, 85, 100%). Telomere peptide nucleic acid probe (TelC-Cy3; PANAGENE) was applied at a final concentration of 4 nM in hybridization buffer (10 mM Tris-HCl [pH 7.5], 70% deionized formamide, 0.5% Roche blocking reagent) and denatured on slide at 75°C for 1 min. Hybridization was allowed to take place for 1.5 h at RT or overnight at 4°C, followed by wash buffer 1 (10 mM Tris-HCl, 1 mg/ml BSA, 70% formamide) two times for 15 min and wash buffer 2 (100 mM Tris-HCl [pH 7.5], 150 mM NaCl, 0.08% Tween 20) three times for 5 min. Slides were dehydrated again in ethanol series and air dried before mounting a glass cover slip using ProLong Gold mounting medium with DAPI (ThermoFisher). DNA-FISH for *Pax5* and *Foxp2* was performed on Interphases using custom-made dual-color break-apart probes (Agilent), as previously described (21). Red and green signals relate to Agilent fluorochromes with ex 547nm/ em 565nm (orange-red) and ex 495nm/ em 517nm (green). Detailed information on custom-made DNA-FISH probes is provided in Supplemental Fig. 4C.

### Microscope image acquisition and automated analysis

Imaging of immunofluorescence and in situ hybridization was carried out using a Nikon Eclipse E400 wide-field microscope equipped with a Hamamatsu Orca camera using a 100× oil-immersion objective. Acquisition was controlled with Micromanager software (23), and images were analyzed using Fiji/ImageJ (24) and CellProfiler (25) for automated counting of native BrdU foci.

### Alkaline comet assay

Two thousand treated cells were mixed with 100 μl of 1% low melting agarose, placed on comet slides (Trevigen), and lysed overnight in prechilled 2.5 M NaCl, 100 mM EDTA, 10 mM Tris, 1% Triton X-100, 10% DMSO, and pH 10 at 4°C. DNA was then allowed to unwind in alkaline solution (300 mM NaOH, 1 mM EDTA, pH >13) for 1 h before electrophoresis at 20 V and 300 mA for 20 min at 4°C. Slides were neutralized in 0.4 M Tris (pH 7.5) for 15 min, fixed in 100% ethanol for 10 min, and air dried. Images of 100 CYGREEN (Enzo Life Sciences)–stained cells were analyzed from duplicate slides of each condition by ImageJ software. All steps were performed in the dark.

### Statistical analysis

Statistical analysis was performed for each experiment as indicated in the figure legends using GraphPad Prism software.

## Results

### SSB2 is dispensable for murine B cells

**FIGURE 1.**
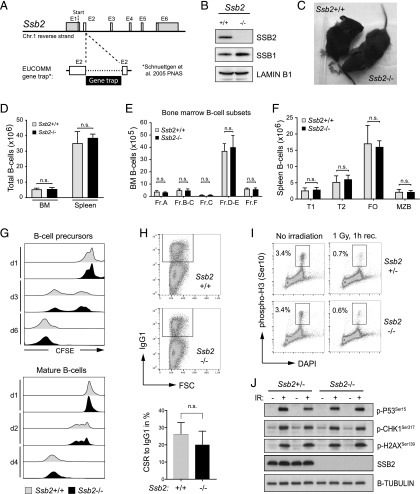
SSB2 function is dispensable for murine B cells. SSB2-deficient mice were generated from targeted ES cells, and B cell compartments and ex vivo–cultured cells from these mice were analyzed for alterations in development, proliferation, and DDR activation. (**A**) Scheme visualizing the targeted disruption of the murine *Ssb2* gene using the GT technique. Integration of the GT cassette into exon 2 disrupts the *Ssb2* open reading frame (see also Supplemental Fig. 1). (**B**) Western blot of whole-cell lysates from mature B cells, confirming SSB2 protein loss in *Ssb2*^−/−^ mice. (**C**) Image of *Ssb2*^−/−^ and wild-type mice. (**D**) Bar diagram showing absolute numbers of CD19^+^ B cells from BM and spleens of *Ssb2*^−/−^ (black) and wild-type (gray) mice determined by flow cytometry (*n* = 3). (**E** and **F**) Bar diagrams showing absolute numbers of BM (E) and splenic (F) B cell compartments in *Ssb2*^−/−^ and wild-type mice (*n* = 3) defined by flow cytometric analysis of BM and spleens. Error bars indicate SEM. BM B cell compartments are those defined by Hardy and Hayakawa (49) and characterized by surface expression of B220, CD19, CD24, and CD43. Splenic B cell fractions were defined by CD19, CD21, and CD23. Gating strategies and backgating to define B cell subsets are shown in Supplemental Fig. 1D, 1E. (**G**) Representative images of CFSE-stained, ex vivo–cultured, and IL-7–stimulated BM B cells (upper panel) and of LPS/IL-4–stimulated splenic B cells (lower panel) from *Ssb2*^−/−^ and wild-type mice (*n* = 3). The days of analysis post-CFSE labeling are indicated. (**H**) Analysis of NHEJ-dependent Ig CSR toward IgG1 in LPS/IL-4–stimulated splenic B cells from *Ssb2*^−/−^ and wild-type mice determined by flow cytometry. Representative dot plots (upper panel) and a bar diagram (lower panel, *n* = 4) are shown. (**I**) Analysis of DNA damage–induced G_2_/M cell cycle arrest in irradiated splenic B cells from *Ssb2*^−/−^ mice (1 Gy, 1 h recovery) by flow cytometric detection of intracellular serine 10–phosphorylated Histone 3. (**J**) Western blot analysis of DNA damage–induced p-P53^Ser15^, p-CHK1^Ser317^, and p-H2AX^Ser139^ using whole-cell lysates from LPS/IL-4–stimulated splenic B cells (IR: 5 Gy, 1 h recovery). For (I) and (J), cells from *Ssb2*^+/−^ mice were used as Ctrl, and representative images of three independent experiments are shown.

To characterize the relevance of the SSB proteins for B cell development, we aimed to generate a mouse model that allows the analysis of combined loss of *Ssb1* and *Ssb2* function. To do so, we first generated *Ssb2*^−/−^ mice to combine this genetic strain with our previously described conditional *Ssb1* KO mouse model (12). Targeted ES cells from the European Conditional Mouse Mutagenesis Program were used to generate *Ssb2*^−/−^ mice. These cells contain a disrupted *Ssb2* locus with a GT cassette (20) inserted into the open reading frame of exon 2, thus abrogating its expression irrespective of cassette orientation (Fig. 1A, Supplemental Fig. 1A). Homozygous *Ssb2*^−/−^ mice were generated from ES-derived *Ssb2*^+/−^ mice, and ablation of SSB2 protein in these mice was verified in splenic B cells by Western blotting (Fig. 1B). As expected from our previous study, SSB2 loss led to a small but visible increase in SSB1 protein levels (Fig. 1B). Unlike *Ssb1*^−/−^ mice generated by others and us (10–12), *Ssb2*^−/−^ mice were viable and did not show any obvious phenotypic developmental abnormalities (Fig. 1C). We next analyzed the composition of T cell compartments in the thymus and B cell compartments in the BM and spleen using flow cytometry. In agreement with a previous study (26), we did not observe any abnormalities in T cell compartments in *Ssb2*^−/−^ mice (Supplemental Fig. 1B, 1C). SSB2 loss also did not affect the composition of early B cell compartments in the BM or mature B cell compartments in the spleen (Fig. 1D–F). We next isolated immature B cells from the BM and mature B cells from the spleen of *Ssb2*^−/−^ and wild-type mice and analyzed them for alterations in cell division during ex vivo cell culture using CFSE labeling and flow cytometry. CFSE-labeled cells reduce intracellular CFSE dye concentration each cell division, and reduction of CFSE is used for the analysis of proliferation. Cell division and survival of ex vivo–cultured BM B cells were promoted by the addition of IL-7, whereas proliferation of mature B cells from the spleen was induced by the addition of IL-4 and LPS. Ex vivo–cultured immature and mature B cells showed normal proliferation in the absence of SSB2 (Fig. 1G). SSB2 loss also did not significantly affect (Ig) CSR toward IgG1 in LPS/IL-4–stimulated mature B cells (Fig. 1H). Similar to our previous observations in *Ssb1*-deficient mice (12), mature B cells from *Ssb2*^−/−^ mice further showed functional canonical G_2_/M cell cycle arrest (indicated by loss of H3 serine 10 phosphorylation, Fig. 1I) and phosphorylation of DDR proteins (Fig. 1J) upon ionizing IR. Hence, we conclude that SSB2 is dispensable for the development of mouse B cells and their capacity to trigger the DDR.

### Combined loss of SSB1 and SSB2 impairs embryogenesis

**FIGURE 2.**
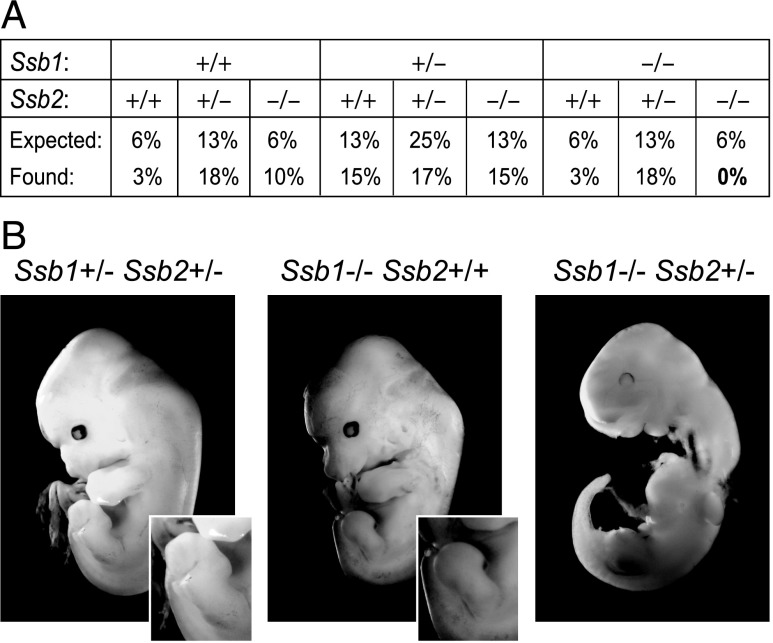
Combined loss of SSB1 and SSB2 impairs embryogenesis. The effect of combined loss of SSB1/2 protein on embryonic development was monitored by analysis of embryos at midgestation. *Ssb1*^+/−^*Ssb2*^+/−^ mice were interbred and analyzed for their offspring at gestation days E10.5–E13.5. A total of 60 embryos were analyzed. (**A**) Summary of the embryo genotypes observed. (**B**) Representative images of *Ssb1*^+/−^*Ssb2*^+/−^, *Ssb1*^−/−^*Ssb2*^+/+^, and *Ssb1*^−/−^*Ssb2*^+/−^ embryos. Insets indicate normal hind limbs in *Ssb1*^+/−^*Ssb2*^+/−^ embryos and early-stage hind limb defects in *Ssb1*^−/−^*Ssb2*^+/+^ embryos, as described (12).

We next interbred double heterozygous *Ssb1*^+/−^;*Ssb2*^+/−^ mice and analyzed their offspring (Fig. 2). Because *Ssb1* loss causes perinatal lethality, we analyzed embryos at gestation days embryo (E) 10.5–E13.5. As expected, *Ssb1*^+/+^;*Ssb2*^−/−^ embryos appeared normal, and *Ssb1*^−/−^;*Ssb2*^+/+^ embryos exhibited early signs of their typical hind limb defect (12) (Fig. 2B). Additional loss of one *Ssb2* allele in *Ssb1*^−/−^;*Ssb2*^+/−^ embryos resulted in defects in the developing embryo (Fig. 2B). In contrast, most *Ssb1*^+/−^;*Ssb2*^−/−^ embryos were normal, although occasional developmental abnormalities with variable penetrance were observed (three of nine embryos with abnormal development). Viable *Ssb1*^+/−^*Ssb2*^−/−^ mice showed no obvious phenotype compared with wild-type mice. This is in agreement with the general assumption that SSB1 function is superior to SSB2 function. Within 60 pups analyzed between gestation days E10.5–E13.5, we did not identify embryos with an *Ssb1*^−/−^;*Ssb2*^−/−^ genotype (Fig. 2A), indicating a minimal requirement for either SSB1 or SSB2 protein during early embryogenesis. We also analyzed normal and abnormal embryos by Western blot for DNA damage–associated phosphorylation of P53 at serine 15 (p-P53^Ser15^), which can be detected in malformed developing hind limbs of *Ssb1*^−/−^ embryos (12). However, abnormal *Ssb1*^−/−^;*Ssb2*^+/−^ embryos did not show elevated p-P53^Ser15^ (Supplemental Fig. 2), suggesting that, if DNA damage was causing the observed malformation, it must have occurred prior to the E10.5–E13.5 gestation period.

### B cell–specific SSB1/2 loss impairs early B cell development

**FIGURE 3.**
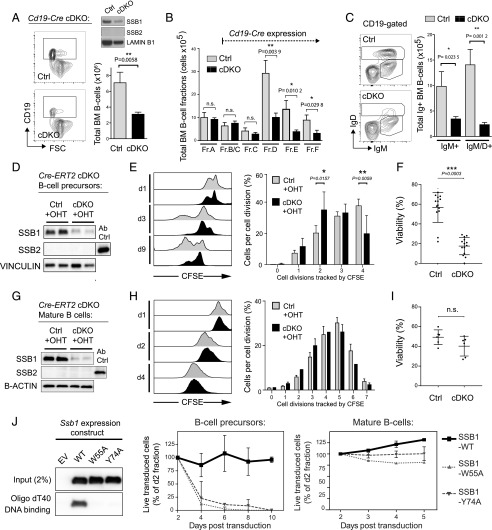
Combined *Ssb1*/*2* deficiency impairs early B cell development and is differentially tolerated by immature and mature B cells. (**A**–**C**) Single cells from BM of *Cd19Cre*/^+^ cDKO mice (*Ssb1flox/flox*;*Ssb2*^−/−^;*Cd19Cre*/^+^ or *Ssb1flox*/^−^;*Ssb2*^−/−^;*Cd19Cre*/^+^) and Ctrl mice (*Ssb1*^+/+^;*Ssb2*^−/−^;*Cd19Cre*/^+^) were analyzed by flow cytometry. (A) Representative flow cytometry plots (left) and a bar diagram indicating total BM B cell numbers (right) in *Cd19Cre*/^+^ cDKO and Ctrl mice are shown (Ctrl, *n* = 4 and cDKO, *n* = 6 mice from >3 analyses). The inset Western blot indicates reduced SSB1 protein levels in BM B cells from *Cd19Cre*/^+^ cDKO mice. (B) Absolute numbers of BM B cell compartments in *Cd19Cre*/^+^ cDKO and Ctrl mice are shown (cDKO, *n* = 6 and Ctrl, *n* = 4; gating strategies and backgating to define B cell subsets are shown in Supplemental Fig. 1D, 1E). (C) Representative dot blot images (left) and bar diagrams (right) showing absolute numbers of BM B cells with or without expression of sIg (IgM^+^ and IgM/D^+^) (DKO, *n* = 6 and Ctrl, *n* = 4). (**D**–**F**) Analysis of IL-7–cultured BM B cells from *Cre-ERT2* cDKO (*Ssb1flox/flox*;*Ssb2*^−/−^;*Cre-ERT2*) and Ctrl mice (*Ssb1*^+/+^;*Ssb2*^−/−^;*Cre-ERT2*). 4-hydroxytamoxifen (OHT) was used to induce CRE recombinase activation and DKO induction. (D) Representative Western blots are shown, confirming DKO induction. Wild-type B cells were blotted as positive Ctrl for the anti-SSB2 Ab. (E and F) Analysis of proliferation and viability, respectively. (E, left) Representative histograms of CFSE-stained BM B cells are shown with time points of analysis indicated. Bar diagrams indicate proliferation (E) and viability (F) at day 6 after OHT addition (*n* = 14 mice per group analyzed in >3 experiments). (**G**–**I**) Analysis of LPS/IL-4–cultured splenic B cells from *Cre-ERT2* cDKO and Ctrl mice as in (D)–(F). (G) Representative Western blots are shown confirming OHT-induced SSB1/2 loss in splenic B cells. Diagrams in (H) and (I) indicate proliferation and viability as in (E) and (F) at day 3 after OHT addition and represent *n* = 6 (Ctrl) and *n* = 8 mice (cDKO) analyzed in >3 experiments. (H, left) Representative histograms of CFSE-stained splenic B cells are shown, with time points of analysis indicated. Error bars in (A)–(F) indicate SEM; statistical analysis was performed using two-tailed *t* test. (**J**, left) Representative Western blot images are shown, confirming the inability of the SSB1-W55A and SSB1-Y74A mutants to bind ssDNA, as described (10). B cell precursors (middle) and mature-B cells (right) from BM and spleens of *Ssb2*^−/−^ mice were transduced with SSB1-WT, SSB1-W55A, and SSB1-Y74A encoding retrovirus (RV), and percentages of live transduced cells were monitored by flow cytometry for RV-encoded GFP expression at indicated time points (*n* = 4 experiments, error bars indicate SD, statistical analysis was performed using the two-tailed *t* test).

To investigate the importance of SSB1/2 function for murine B lymphocytes and circumvent embryonic lethality caused by *Ssb1* loss (11, 12), we next generated SSB1/2 conditional KO mice, in which combined SSB1 and SSB2 loss in B cells is mediated via the *Cd19*-*Cre* transgene (cDKO, *Ssb1flox/flox*;*Ssb2*^−/−^;*Cd19Cre*/^+^). CD19 is exclusively expressed by B cells, hence *Cd19* promoter-driven *Cre*-recombinase expression in cDKO mice induces a combined SSB1/2 deficiency limited to B cells. As SSB2-deficient B cells did not differ from wild-type B cells in our model (Fig. 1), we analyzed *Ssb1*^+/+^;*Ssb2*^−/−^;*Cd19Cre*/^+^ mice as Ctrl. Analysis by flow cytometry revealed an ∼55% decrease in B cells in the BM of *Cd19*Cre cDKO compared with Ctrl mice (Fig. 3A). A more-detailed analysis of individual BM B cell compartments showed that cDKO mice exhibit a specific reduction of cells at the pre-B to immature B stage and consecutive stages of B cell differentiation (fraction D–F, Fig. 3B), mirrored by reduced numbers of IgM- and IgM/D–expressing B cells in the BM of cDKO mice (Fig. 3C). As a consequence, numbers of mature B cells in the spleen were reduced and spleen sizes decreased in cDKO mice compared with Ctrl (Supplemental Fig. 3A, 3B). Intriguingly, we observed that mature B cells in the spleen of our *Cd19Cre* cDKO model had largely restored SSB1 expression (Supplemental Fig. 3C). This is likely caused 2by positive selection for cDKO cells with silenced *CD19-Cre* transgene expression during early B cell development, reflecting the importance of SSB proteins for normal B cell development.

### Loss of SSB1/2 alters proliferation and survival of B cell precursors but is largely tolerated by mature B cells

To overcome the limitations of our *Cd19Cre* cDKO model and investigate SSB1/2 function in B cells in more depth, we next generated mice in which combined loss of SSB1/2 can be induced via a Tamoxifen (OHT)-inducible transgene (*Cre-ERT2*) (*Ssb1flox/flox*;*Ssb2*^−/−^;*Cre-ERT2*, Fig. 3D, 3G). *Ssb1*^+/+^;*Ssb2*^−/−^;*Cre-ERT2* mice were analyzed as Ctrl. As we observed a defect on early B cell development in our *Cd19Cre* cDKO model, we first investigated the effect of OHT-induced SSB1/2 depletion on B cell precursors. Cells were isolated from the BM of *Cre-ERT2* cDKO and Ctrl mice and propagated by ex vivo culture in the presence of IL-7. This culture condition allows selective propagation of CD43^+^CD24^+^IgM^−^ pro/pre–B cells (27), with IL-7 inducing proliferation and survival and indirectly suppressing the RAG recombinases required for V(D)J recombination (28, 29). Western blotting confirmed depletion of SSB1 and SSB2 protein levels in OHT-treated *Cre-ERT2* cDKO cells (Fig. 3D). In line with our in vivo results, SSB1/2 loss in ex vivo–cultured cycling B cell precursors caused a significant delay in proliferation (Fig. 3E) and a concomitant decrease in viability (Fig. 3F). We next investigated whether SSB1/2 loss might also impair B cell differentiation, which depends on successful DNA recombination of the Ig loci. We applied an experimental approach that is based on withdrawal of IL-7 from IL-7 cultures and concomitant provision of BAFF (27), which leads to the emergence of surface Ig (sIg) positive cells from sIg-negative cultures. We also assessed the consequences of IL-7 withdrawal combined with LPS treatment, which has been used to enhance RAG expression (30, 31). Before IL-7 withdrawal, we depleted residual sIg^+^ cells (∼1–2%) from our culture by magnetic cell sorting, and SSB1/2 loss was induced by OHT treatment (Supplemental Fig. 3E). Cell cultures were monitored for presence of sIg^+^ cells (i.e., IgM/IgK^+^) by flow cytometry 3 and 4 d after IL-7 withdrawal. SSB1/2 loss led to minor reductions in sIg^+^ cells under IL-7-withdrawal conditions (Supplemental Fig. 3F, 3G). Notably, SSB1/2 loss induced only negligible levels of cytotoxicity in the absence of IL-7–induced proliferation (Supplemental Fig. 3H).

We next analyzed the effect of SSB1/2 loss on mature B cells using splenocytes from *Cre-ERT2* cDKO and Ctrl mice. Splenic B cells were propagated ex vivo by cell culture in the presence of LPS and IL-4 to induce proliferation and Ig CSR, and SSB1/2 loss was induced by OHT treatment (Fig. 3G). Surprisingly, proliferating mature B cells tolerated SSB1/2 loss and did not show significant differences in proliferation and survival compared with Ctrl cells (Fig. 3H, 3I). Likewise, Ig CSR was only minimally affected by SSB1/2 loss (Supplemental Fig. 3D). To validate the differential sensitivity of immature compared with mature B cells with a different methodological approach, we made use of SSB1 ssDNA-binding mutants (W55A and Y74A) previously described by others (10). Although these mutant proteins fail to bind ssDNA (Fig. 3J, left), they are still able to interact with the SSB1/2 complex-forming protein INTS3 (10) and are thereby likely to exhibit a dominant-negative effect on the function of both SSB proteins. In agreement with our model, primary IL-7–cultured BM B cell precursors from *Ssb2^−/−^* mice were highly sensitive to SSB1-W55A or -Y74A expression (Fig. 3J, middle), whereas their expression had no effect on primary LPS/IL-4–cultured mature splenic B cells from *Ssb2^−/−^* mice (Fig. 3J, right).

### Combined genetic loss of both SSB proteins can be rescued by expression of either SSB1 or SSB2 and suppressed by BCL2

**FIGURE 4.**
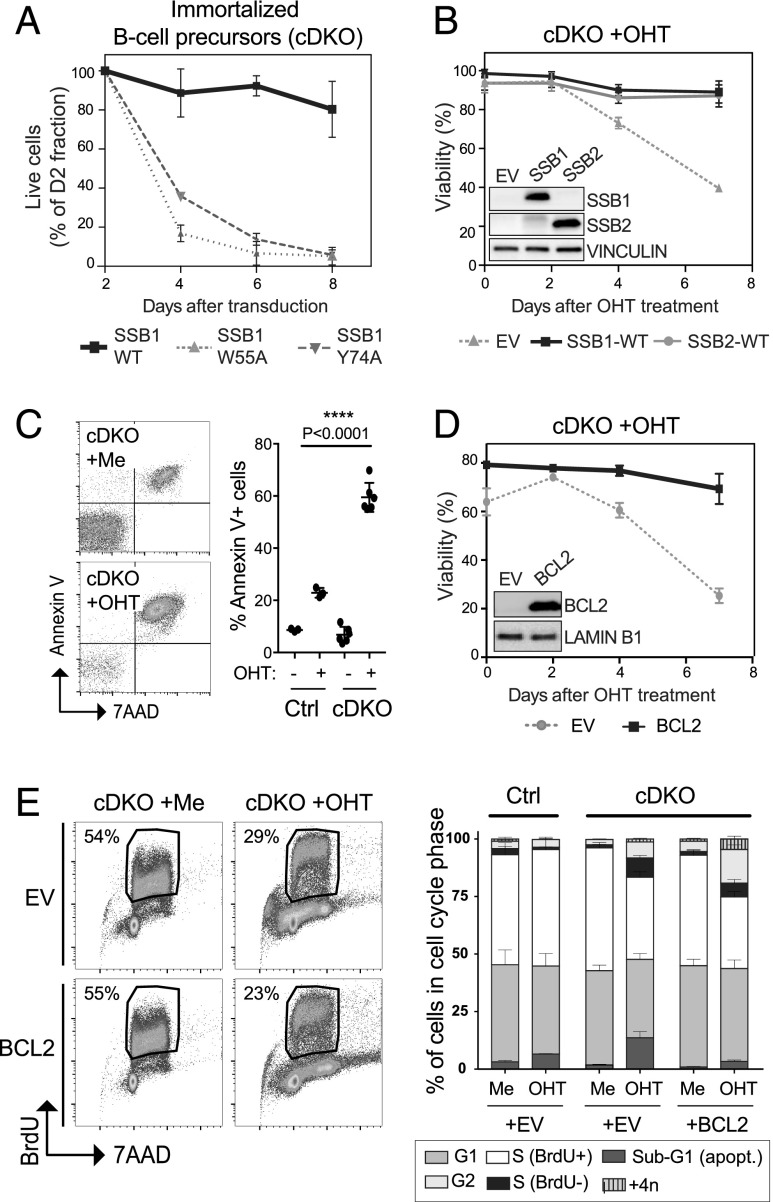
Genetic loss of both SSB proteins can be rescued by overexpression of either SSB1 or SSB2 and suppressed by expression of BCL2 and alters cell cycle progression. Primary B cell precursors from *Cre-ERT2* cDKO mice (*Ssb1flox/flox*;*Ssb2*^−/−^;*Cre-ERT2*) and Ctrl mice (*Ssb1*^+/+^;*Ssb2*^−/−^;*Cre-ERT2*) were immortalized by ectopic expression of c-MYC and used for the analysis of SSB1/2 loss on survival or cycle progression. (**A**) Immortalized cDKO B cell precursors were transduced with SSB1-WT, SSB1-W55A, or SSB1-Y74A encoding retrovirus (RV), and percentages of live transduced cells were monitored by flow cytometry for RV-encoded DSRED expression. (**B**) Immortalized cDKO B cell precursors were stably transduced with wild-type SSB1 or SSB2 encoding RV, or EV as Ctrl, and viability was measured at indicated time points after OHT-induced DKO induction. The inset shows a representative Western blot confirming DKO induction and forced expression of SSB1-WT and SSB2-WT in OHT-treated DKO cells. (**C**) Analysis of OHT- and mock-treated (MeOH) cDKO and Ctrl cells by flow cytometry using Annexin V and 7-AAD dyes. Representative dot blots (left) and a bar diagram (right) summarizing the results of *n* = 3 experiments are shown using one Ctrl and two independent cDKO cell lines each experiment. (**D**) Viability of DKO cells ectopically expressing BCL2 measured at indicated time points after the start of OHT treatment. EV-transduced DKO cells were used as Ctrl. (**E**) Cell cycle distribution of BrdU pulse-labeled and 7-AAD–labeled OHT- and mock-treated cDKO, Ctrl, and BCL2-expressing cells determined by flow cytometry. Left, Representative dot plots of BrdU/7-AAD staining. Right, Bar diagram indicating fractions of cells in individual cell cycle stages for the conditions indicated. Data represent *n* = 4 experiments using one Ctrl and two independent cDKO cell lines for each experiment. For experiments shown in (C) and (E), cells were treated for 2–3 d with OHT, reincubated in normal medium, and analyzed on day 5 post-OHT addition. For experiments shown in (B) and (D), analysis was performed at time points as indicated. Error bars indicate SD, and statistical analysis was done using two-tailed *t* test.

To investigate the detrimental effect of SSB1/2 loss in B cell precursors in more detail, we next generated immortalized B cell precursors from *Cre-ERT2* cDKO and Ctrl mice using c-MYC–encoding retrovirus, as described (21). Similar to primary B cell precursors, MYC-immortalized cDKO B cell precursors were sensitive to SSB1-W55A and -Y74A expression (Fig. 4A) and OHT-induced SSB1/2 depletion (EV cells in Fig. 4B). OHT-induced SSB1/2 deficiency could be rescued by ectopic expression of either SSB1 or SSB2 (Fig. 4B), demonstrating the redundant roles of the two proteins for cell survival. To determine whether OHT-induced SSB1/2 depletion causes apoptosis, we next analyzed OHT-treated cDKO and Ctrl cells by flow cytometry using Annexin V and 7-AAD staining. MeOH-treated cells were analyzed as Ctrl. OHT-treated cDKO cells became positive for both Annexin V and 7-AAD upon SSB1/2 loss (Fig. 4C), thereby precluding differentiation between apoptosis and necrosis. OHT treatment also had a temporary effect on our Ctrl cells during the time of OHT treatment. However, whereas Ctrl cells recovered upon OHT removal, the viability of cDKO cells continued to decrease at consecutive days (Fig. 4C). To assess the cause of cell death with a different approach, we next investigated whether the cytotoxicity induced by SSB1/2 loss could be rescued by ectopic expression of the antiapoptotic protein BCL2. Indeed, BCL2 expression largely rescued OHT-induced SSB1/2 depletion, thereby indicating that SSB1/2 loss is triggering apoptosis (Fig. 4D).

As hSSB1 proteins were shown to be able to substitute for RPA1 in ATRIP-ATR recruitment (32), we further assessed whether elevated RPA expression might in turn be able to compensate for SSB1/2 loss. Of note, whereas RPA is only functional if all three subunits are expressed and rate limiting during replication stress (33), B cell precursors seem to express very low levels of RPA2 (Supplemental Fig. 4A). We therefore ectopically expressed the RPA1-RPA2-RPA3 complex in a stoichiometric fashion in immortalized cDKO cells using a construct that expresses all three RPA subunits on a single transcript [i.e., superRPA/sRPA (33)] (Supplemental Fig. 4B, left). However, forced expression of sRPA did not rescue OHT-induced SSB1/2 loss (Supplemental Fig. 4B, right).

### SSB1/2 loss alters cell cycle progression, induces nuclear ssDNA exposure, and disrupts genome fragile sites

**FIGURE 5.**
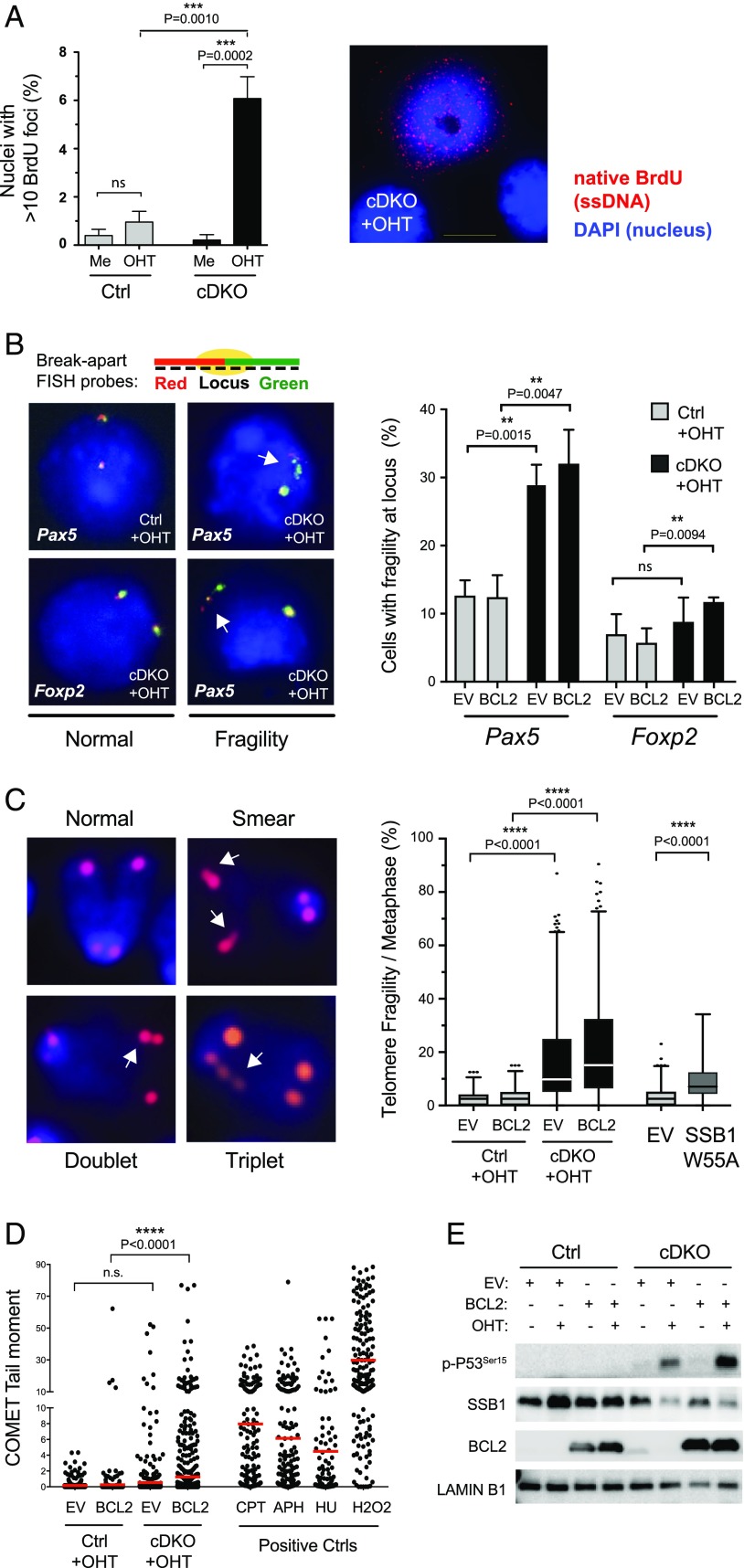
SSB1/2 loss in B cell precursors increases nuclear ssDNA exposure, disrupts genome fragile sites, and causes DNA damage if apoptosis is suppressed. Immortalized cDKO (*Ssb1flox/flox*;*Ssb2*^−/−^;*Cre-ERT2*) and Ctrl (*Ssb1*^+/+^;*Ssb2*^−/−^;*Cre-ERT2*) B cell precursors were analyzed for nuclear ssDNA exposure, fragile site disruption, and ssDNA breaks upon DKO induction. (**A**) BCL2-expressing cDKO and Ctrl cells were OHT- or mock-treated for 2 d, reincubated in normal medium, and BrdU labeled. Images of extracted nuclei stained under nondenaturing conditions using anti-BrdU Abs were taken 4 d after the start of experiment. A bar diagram representing *n* = 5 experiments (>100 nuclei per experiment) (left) and a representative image of a cell with >10 native BrdU foci are shown (right). (**B**) DNA-FISH analysis of the B cell–specific fragile site *Pax5* and a nonfragile locus (*Foxp2*) using locus-specific break-apart probes. For each locus, red- (excitation 547 nm, emission 565 nm) and green-labeled (excitation 495 nm, emission 517 nm) probes were used that cover individual parts of the locus and yield in one yellow signal if the locus is intact. Analysis was performed on interphases of OHT-treated cDKO and Ctrl cells, treated as described in Fig. 4E. Left, Representative images of nuclei with intact and disrupted loci are shown. Disrupted loci are indicated by white arrows. Right, Fractions of interphases with nonintact loci were counted for the conditions indicated (*n* = 3 experiments with at least 150 interphases per condition and experiment; error bars represent SD, unpaired student’s *t* test was employed to test significance). (**C**) DNA-FISH analysis of telomere integrity using cyanine 3 (Cy3)-labeled telomere-specific probes (red) and DAPI (blue). Cells were treated as above, fixed, and metaphase spreads were prepared. Representative images of reported telomere fragility phenotypes, such as smear, doublet, or triplet signals (indicated by white arrows) (37) are shown (left) and summarized for *n* = 4 experiments for the conditions indicated (right). Quantification of % fragility per metaphase is presented as box plot of interquartile range; bars are defined as range from 0 to 99%, with the highest values of the data set depicted as dots. At least 40 metaphases per condition and experiment were analyzed. For analysis of SSB1-W55A–expressing cells, 33 metaphases were analyzed, obtained from three experiments. (**D**) Quantification of DNA breakage in OHT-treated cDKO and Ctrl cells by comet assay (*n* = 3). DNA breakage per cell is given as the DNA comet tail moment. One hundred cells per condition were analyzed in each experiment. As positive Ctrl, cells were treated with CPT, HU, or APH. (**E**) Western blot analysis of MYC-immortalized cDKO and Ctrl B cell precursors for serine 15–phosphorylated P53, BCL2, SSB1, and lamin B1 as loading Ctrl. Cells used were stably transduced with BCL2 expression vector or EV and OHT-treated as in Fig. 4E.

Simultaneous loss of SSB1/2 in hematopoietic stem cells (HSCs) causes loss of quiescence and premature entry into the cell cycle (34). To investigate whether SSB1/2 loss causes similar cell cycle deregulation in B cell precursors, we used BrdU pulse labeling to monitor cell cycle progression in SSB1/2-depleted cells (Fig. 4E). We observed that, besides induction of cell death (cells in sub-G_1_), SSB1/2 loss caused a significant reduction of cells that actively incorporated BrdU during S-phase progression (BrdU^+^), accompanied by an accumulation of cells in S phase that did not incorporate BrdU (BrdU^−^). Besides, SSB1/2 loss caused more cells to accumulate in G_2_. Hence, opposite to the effect on HSCs, SSB1/2 loss in B cell precursors perturbs S-phase progression and promotes cell cycle arrest at G_2_. To investigate the underlying reason for this defect in cell cycle progression, we next assessed whether loss of SSB1/2 might cause aberrant ssDNA exposure using nondenaturing BrdU analysis. Unprotected ssDNA is associated with the formation of secondary ssDNA structures, which can interfere with replication and hence could cause the observed defect in cell cycle progression. To prevent quantification of BrdU exposed during apoptosis, we assessed the presence of ssDNA after double KO (DKO) induction in BCL2-expressing cells. In addition, nuclei were extracted preceding our analysis to exclusively visualize nuclear ssDNA. Indeed, we observed increased exposure of nuclear ssDNA in SSB1/2-depleted cells (Fig. 5A). As secondary ssDNA structures can cause disruption of chromosomal fragile sites (reviewed by Ref. 35), we next analyzed whether the integrity of fragile sites is affected upon SSB1/2 loss using DNA-FISH. First, we examined the *Pax5* locus, a B cell–specific early replicating fragile site identified by others and us as instable in mature and immature murine B cells (21, 36). A nonfragile locus (*Foxp2*) (21) was assessed as a Ctrl. SSB1/2 loss indeed caused significant fragility of *Pax5*, which was further elevated in BCL2-expressing DKO cells (Fig. 5B). Fragility at the *Foxp2* locus increased slightly but at much lower levels. We further analyzed the integrity of telomeres, which resemble common fragile sites irrespective of the cell type (37). We quantified all acknowledged types of telomere fragility, including smears, doublets, and triplets in this analysis (Fig. 5C, left). As for *Pax5*, we observed increased fragility of telomeres upon DKO induction, which was further increased in BCL2-expressing DKO cells (Fig. 5C, right). Telomere fragility was also increased in cells expressing the dominant-negative ssDNA-binding mutant SSB1-W55A (Fig. 5C, right). We next analyzed DKO and BCL2-expressing DKO cells using the alkaline single-cell gel electrophoresis assay (i.e., comet assay) to measure DNA single-strand breaks. *Cre-ERT2* cDKO B cell precursors treated with aphidicolin (APH), camptothecin (CPT), hydroxyurea (HU), or hydrogen peroxide (H_2_O_2_) were used as positive Ctrl. SSB1/2 ablation caused a minor increase of DNA damage detectable by the comet assay. However, suppression of apoptosis by forced expression of BCL2 in parallel to DKO induction increased DNA damage to significant levels (Fig. 5D). In line with this, DNA damage–associated p-P53^Ser15^ upon OHT-mediated DKO induction became most apparent in cells ectopically expressing BCL2 (Fig. 5E). As a few thousand ssDNA breaks per cell have been reported to be needed for a detectable signal in the comet assay (38), our results suggest that under normal conditions, apoptosis of B cell precursors upon SSB1/2 DKO induction occurs before this threshold is reached.

## Discussion

SSB1 and SSB2 have been described as mediators of the DDR and guardians of genome stability in response to DNA damage in human cells. However, their requirement for cell viability and genome integrity under normal conditions has been less investigated. We previously observed that RNA interference–mediated downregulation of SSB2 in *Ssb1* monoallelic (*Ssb1flox*/-) SV40-transformed murine embryonic fibroblasts impaired their cell division (12). In line with this, Gu et al. (10) showed that downregulation of SSB2 in *Ssb1^−/−^* SV40-transformed murine embryonic fibroblasts induced lethality, suggesting that minimal SSB protein levels might be vital for life. In contrast, INTS3 depletion in human cell lines, which results in loss of expression of both hSSB1 and hSSB2 (5, 39), has not been reported to cause toxicity or alterations in cell division.

To investigate the general requirement for SSB1/2 proteins in more depth, we and others generated conditional mouse models that allowed combined genetic deletion of *Ssb1* and *Ssb2*. Shi et al. (34) showed that ubiquitous deletion of *Ssb1* and *Ssb2* in adult mice compromises the function of hematopoietic stem and progenitor cells, resulting in rapid HSC exhaustion and consecutive depletion of all hematopoietic lineages. However, it remained unclear whether SSB1/2 depletion also affects more differentiated cells of the hematopoietic lineage, such as lineage-committed B cells. We now addressed this open question by investigating the requirement for SSB1/2 function in B cells, specifically by analyzing BM-derived B cell precursors and mature B cells from the spleen. Our results show that BM B cells are sensitive to SSB1/2 dysfunction, whereas mature B cells from the spleen largely tolerate SSB1/2 loss. In line with this, SSB1/2 ablation in vivo impairs early B cell development at the pre-B to immature B cell stage, affecting all consecutive stages. Although progression at this stage depends on successful rearrangement of the Ig loci by V(D)J recombination, we did not find any indication that SSB1/2 loss significantly hampers Ig rearrangement. For example, the induction of Ig^+^ immature B cells from Ig^−^ pro/pre–B cell cultures is only affected to a minor extent by SSB1/2 loss. Likewise, Ig CSR, which like V(D)J recombination depends on functional repair of DNA DSB by cNHEJ, was not significantly altered by SSB1/2 loss. However, our experiments do not exclude the possibility of subtle effects of SSB1/2 loss on the joining of Ig gene segments (e.g., N nucleotide addition during V(D)J recombination), and thus a more in-depth analysis of V(D)J recombination products in SSB1/2-deficient B cell precursors may still uncover yet-unknown functions of SSB1/2 proteins in V(D)J recombination. Early B cell development is also characterized by clonal expansion of B cell precursors [e.g., upon successful completion of the pre-BCR checkpoint (40); reviewed in Ref. 41], and we show that proliferation and survival of cycling B cell precursors is significantly impaired by SSB1/2 loss, whereas their ablation has little effect on survival in the absence of a proliferation stimulus. Thus, the B cell developmental defect observed in SSB1/2 cDKO mice is likely due to cell death occurring during such phases of clonal B cell expansion.

SSB1/2 are ssDNA-binding proteins. To investigate why SSB1/2 proteins are crucial for proliferation of B cell precursors, we first assessed whether SSB1/2 loss causes increased exposure of nuclear ssDNA. In analogy to Toledo et al. (33) who described that global exhaustion of the ssDNA-binding protein RPA causes nuclear ssDNA exposure, we similarly observed that SSB1/2 loss causes increased exposure of nuclear ssDNA. We further show that ssDNA exposure coincides with altered cell cycle progression, underreplicated DNA (i.e., BrdU-negative cells in S phase), chromosomal fragile site disruption, and DNA damage detectable by comet assay if apoptosis is suppressed. We speculate that the absence of SSB proteins might facilitate the formation of secondary structures, such as R-loops and hairpins at ssDNA exposed during various aspects of DNA metabolism, including transcription and replication. As a consequence, SSB loss would interfere with cell cycle progression and promote the disruption of chromosomal fragile sites. In line with this, loss of the ssDNA-binding protein RPA promotes hairpin formation (42) and R-loop stabilization (43), and SSB1/2 loss in hematopoietic stem and progenitor cells is associated with DNA damage at R-loops (34). Chromosomal fragile sites, in contrast, are enriched for DNA sequences that favorably form hairpins (35) and R-loops (44), and their breakage has been associated with underreplicated DNA (45, 46). Notably, although our findings are distinct to those reported by Shi et al. (34), both studies complement each other to consolidate SSB1/2 proteins as important genome safeguards and highlight their importance for development and function of the hematopoietic niche in the BM.

Although we highlight the importance of SSB1/2 proteins for B cell precursors, we similarly show that their function is less required in activated mature B cells. Tolerance to SSB1/2 loss by mature B cells does not relate to slower cell division as LPS/IL-4–stimulated mature B cells typically proliferate faster than IL-7–cultured B cell precursors. However, mature activated B cells are known to exhibit a higher tolerance to DNA damage compared with precursor B cells (47) and hence may similarly better tolerate defects resulting from SSB1/2 dysfunction. Griffiths et al. (47) related this to increased BCL2 expression in mature B cells, and indeed we found that BCL2 expression attenuates SSB1/2 loss. However, we did not observe increased BCL2 expression in mature compared with precursor B cells under our culture conditions (data not shown). Nevertheless, activated mature B cells have been reported to exhibit high expression of DNA damage repair and response proteins (48), which similarly could attenuate the effect of SSB1/2 loss in our model.

In summary, we used a number of different models to conclude that SSB1 and 2 are essential factors for early B cells but are largely dispensable in mature B cells. Although we did not yet provide an explanation for the tolerance of mature B cells to SSB1/2 loss, this will be the subject of future investigations.

## Supplementary Material

Data Supplement
