# Supplementary material for: SSB1/SSB2 Proteins Safeguard B Cell Development by Protecting the Genomes of B Cell Precursors
Source: J Immunol. 2019 May 13;202(12):3423–33. doi: 10.4049/jimmunol.1801618 (PMC6545462; doi:10.4049/jimmunol.1801618)
Supplement: Data Supplement [file JI_1801618.zip › JI_1801618_Supplemental_Figures_1.pdf]

Figure S1

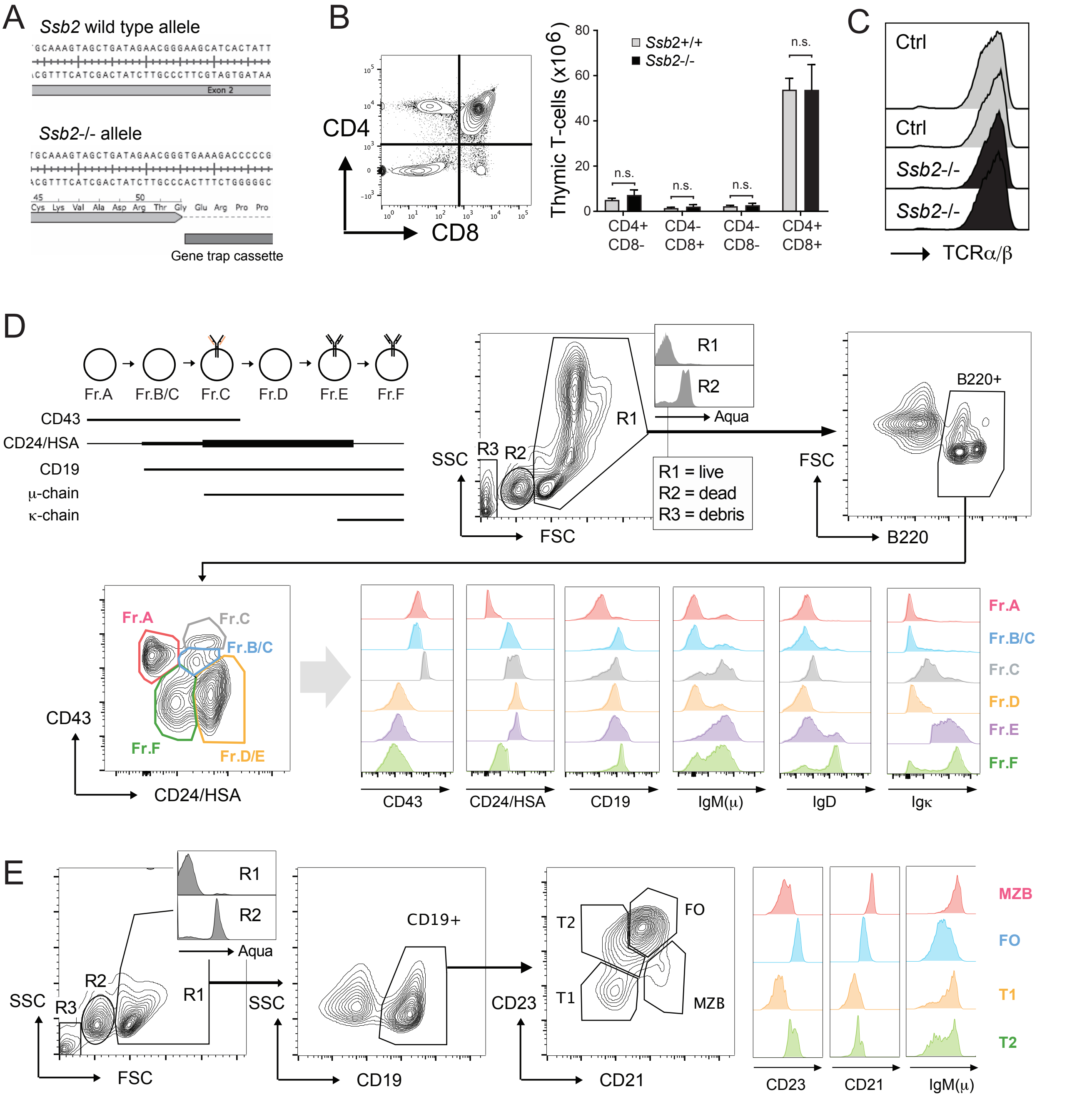

Figure S1: *Ssb2* gene targeting and characterization of B- and T-cell subsets by flow cytometry.
(A) Verification of the gene trap integration site in exon 2 of the targeted *Ssb2* allele by Sanger sequencing. (B/C) Flow cytometry analysis of thymus T-cell subsets of *Ssb2*-/- and wild type mice (n=4) confirming normal T-cell development in *Ssb2*-/- mice as reported by Boucher et al (26) using antibodies for the surface receptors CD3, CD4 and CD8 (B), or the α/β-chains of the T-cell receptor (TCRα/β) (C). (B, left) Flow cytometry gates used to indicate CD4/8-defined T-cell subsets. (D) Strategy for the analysis of bone marrow (BM) B-cell fractions by flow cytometry. (Left, up) Schematic of BM B-cell fractions defined by the expression of CD43, CD24/HSA, CD19, surface Igμ and surface Igκ. Note, that the antibody used by us recognizes the Igμ-chain independent of an Ig light chain (i.e. Igk) and thus recognizes Igμ as part of the pre-B cell receptor (i.e. on Igμ+Igκ- cells) or as part of the B-cell receptor (i.e. surface IgM on Igμ+Igκ+ cells). (Middle, up) Gating strategy to analyse live cells by FSC/SSC gating, live (R1) and dead (R2) cell populations can be distinguished by selective staining by the dead cell dye Aqua (inset). Live cells are further sub-gated for CD19 or B220 (right, up), and then analyzed for CD43 and CD24 expression (left, bottom). Identities of identified BM B-cell fractions were confirmed by back-gating for CD43, CD24/HSA, CD19, Igμ and Igκ expression (histograms) with Fr.A = CD43+CD24^{low}CD19-Igμ-IgD-Igκ-; Fr.B/C = CD43+CD24^{int}CD19+Igμ-IgD-Igκ-; Fr.C = CD43+CD24^{high}CD19+Igμ+IgD-Igκ-; Fr.D/E = CD43-CD24^{high}CD19+Igμ+/-IgD-Igκ+/- and Fr.F CD43-CD24^{low}CD19+Igμ+IgD+Igκ+. In order to distinguish Fr.D and Fr.E BM B-cells (i.e. in Figure 3B), further sub-gating on Igk was performed with Fr.D = CD43-CD24^{high}CD19+Igμ-IgD-Igκ− and Fr.E = CD43-CD24^{high}CD19+Igμ+Igκ+. (E) Strategy for the analysis of splenic B-cell fractions defined by the expression of CD19, CD23 and CD21 by flow cytometry. (Left) Gating strategy to analyse live cells by FSC/SSC gating, live (R1) and dead (R2) cell populations can be distinguished by selective staining by the dead cell dye Aqua (inset) as in (D). Live cells are further sub-gated for CD19 and then analyzed for CD21 and CD23 expression. Identities of identified splenic B-cell fractions were confirmed by back-gating for CD23, CD21 and IgM expression (histograms) with marginal zone B-cells (MZB) = CD23^{low}CD21^{high}IgM^{high}, follicular B-cells (FO) = CD23+CD21^{int}IgM^{int}, transitional 1 B-cells (T1) = CD23^{low}CD21^{low}IgM^{high} and transitional 2 B-cells (T2) = CD23+CD21^{int}IgM^{high}.

Figure S2

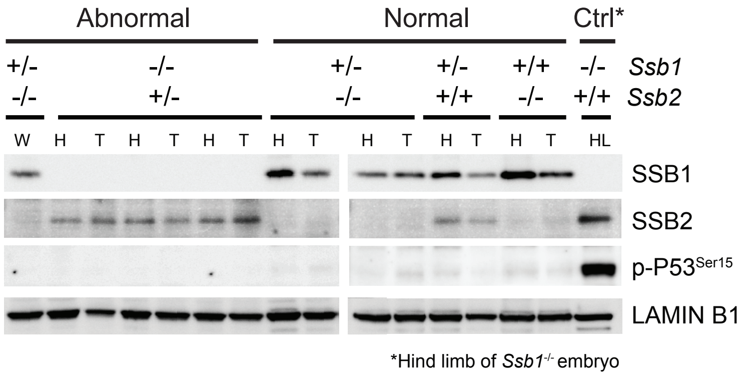

Figure S2: Analysis of abnormal embryos from *Ssb1*+/-*Ssb2*+/- interbreeding by Western blot. Western blot analysis of different compartments of normal and abnormal embryos is shown (W = whole embryo, H = head, T = thorax). Antibodies against SSB1 and SSB2 were used for confirmation of the genotypes, serine 15-phosphorylated P53 (p-P53^{Ser15}) was assessed as an indicator of DNA damage, LAMIN B1 was visualized as loading control. Protein lysate of hind limbs (HL) from E13.5 *Ssb1*-/- embryos was used as a positive control for p-P53^{Ser15} signals.

Figure S3

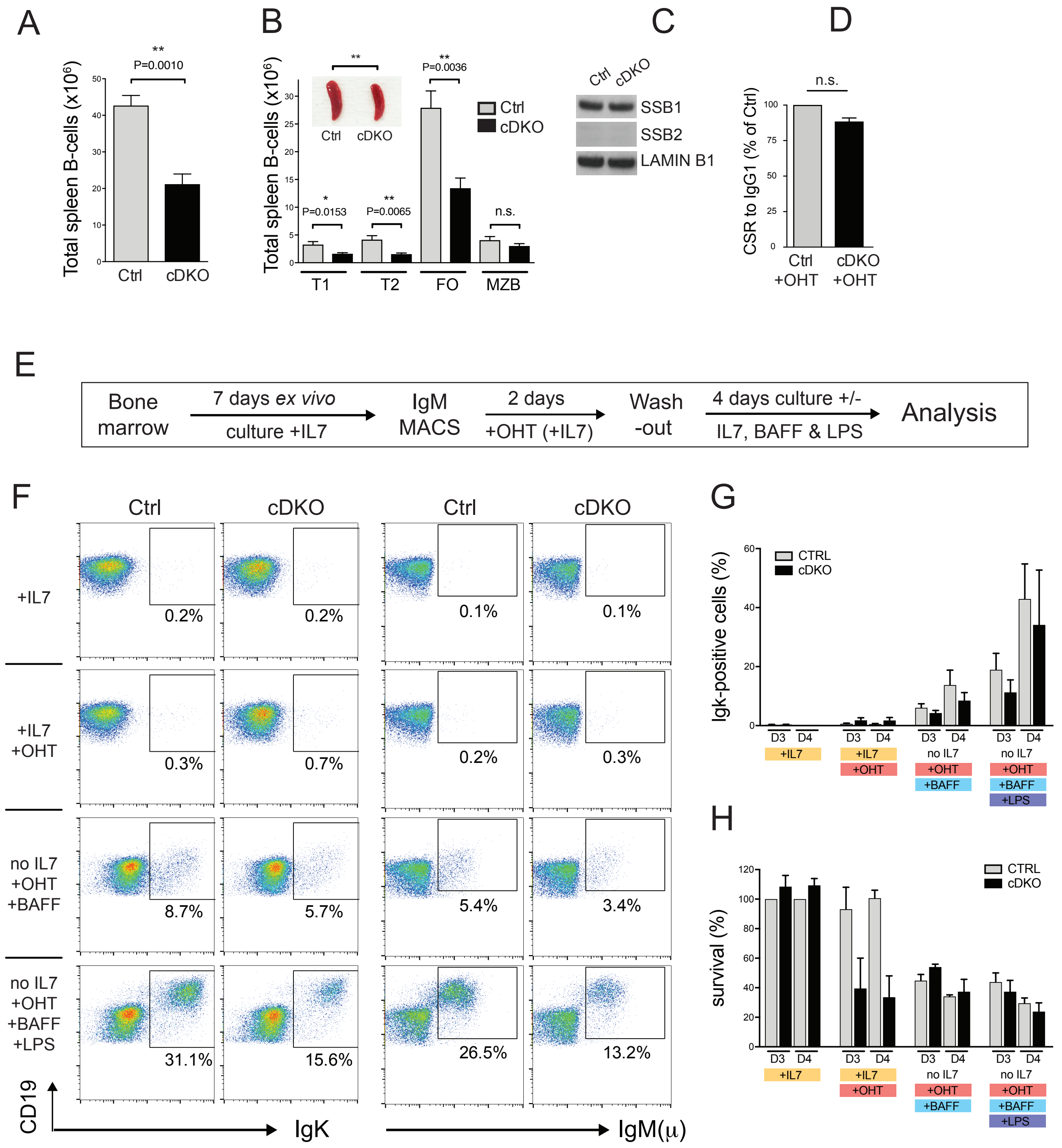

Figure S3: Analysis of B-cells from *Cd19-Cre* cDKO (A-C) mice and *Cre-ERT2* cDKO (D-H) mice.
(A-C) Analysis of B-cells from *Ssb1*flox/flox;*Ssb2*-/-;*Cd19Cre*/+ mice (cDKO n=5, Ctrl n=4). (A/B) Bar diagrams show total numbers of CD19+ splenic B-cells (A) and of CD19+ B-cell subsets (B) determined by flow cytometry as described in Supplementary Figure S1E. Inset shows representative spleen images from *Cd19Cre* cDKO and Ctrl mice. (C) Western blot image for SSB1 protein in splenic B cells from *Cd19Cre* cDKO and Ctrl mice is shown.(D-I) Analysis of B-cells from *Ssb1*flox/flox;*Ssb2*-/-;*Cre-ERT2* mice. (D) Splenic B cells from *Cre-ERT2* cDKO and Ctrl mice were cultured *ex vivo* with LPS/IL4 and monitored for Ig class-switch recombination towards IgG1 by flow cytometry (n=3). (E) Graphical scheme of IL7 withdrawal experiment. Bone marrow from *Cre-ERT2* cDKO and Ctrl mice was cultured *ex vivo* in presence of IL7 for 7 days. On day 7, cells were depleted from residual IgM+ cells (~1-2%) using MACS and OHT treated (2 days, 0.125μM). OHT and IL7 were then washed out and cells plated in media containing IL7, BAFF or BAFF and LPS as indicated. On day 3 and 4, cells were analysed for emergence of IgM+ and IgK+ cells by flow cytometry. (F) Representative flow cytometry plots from day 4 of the experiment described in (E). Cells were pre-gated for live cells using FSC/SSC and Aqua dye and for CD19. (G) Bar diagram summarizing flow cytometric analysis of IgK+ cells for n=2 experiments as described in (E) and the conditions indicated. (H) Bar diagram as in (G) summarizing percentages of live cells defined by using Aqua dye and flow cytometry for n=2 experiments as described in (E) and the conditions indicated.

Figure S4

A

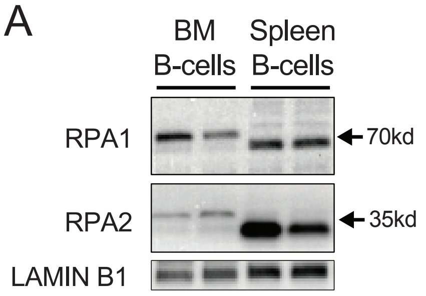

B

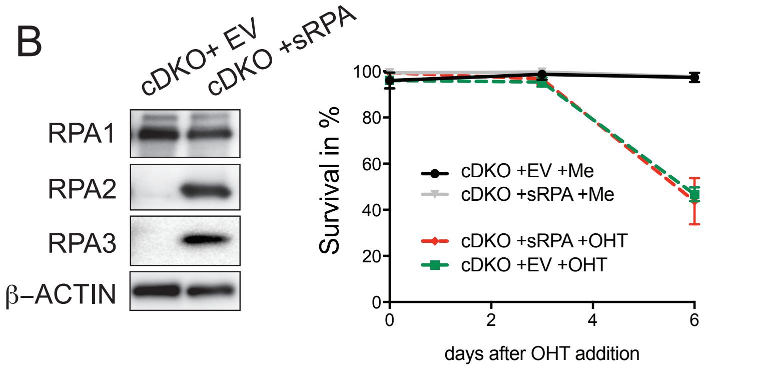

C

***FOXP2* break-apart probe:**

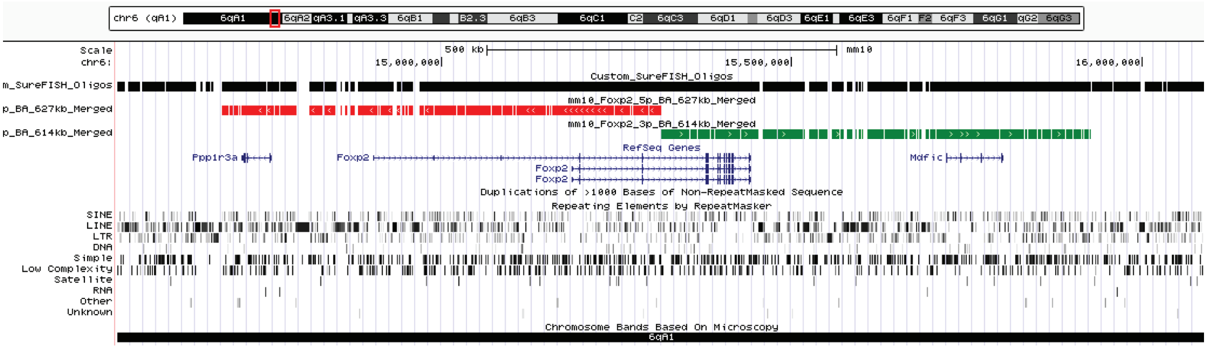

***PAX5* break-apart probe:**

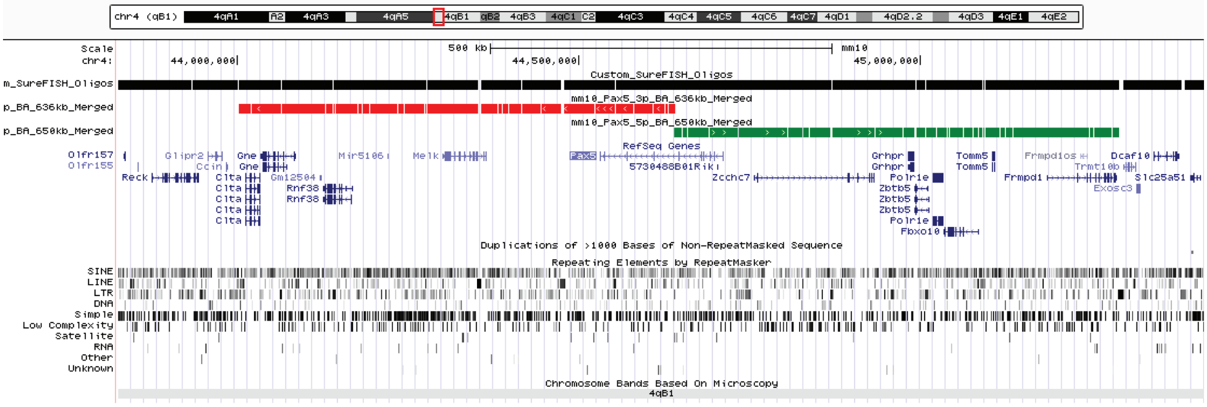

Figure S4: Super-RPA expression does not compensate for SSB1/2 loss.
(A) Western blot for endogenous RPA1 and RPA2 in lysates from IL7-cultured precursor B-cells from the bone marrow and LPS/IL4-cultured mature B-cells from the spleen. Similar results were obtained for OHT-treated cDKO cells (not shown).
(B) Immortalized *Cre-ERT2*+ cDKO B-cell precursors as described in Figure 4 were stably transduced with super-RPA (sRPA) encoding retrovirus, which allows stoichiometric expression of all three RPA subunits of replication protein A (RPA) (29). Empty vector (EV)-transduced immortalized Cre-ERT2+ cDKO cells were used as controls. (Left) Forced expression of all RPA subunits was confirmed by Western blot. (Right) SSB1/2 loss was induced by 3 days of OHT treatment and viability monitored at indicated time points (n=3). (C) Custom designed ‘break-apart’ DNA-FISH probes produced by Agilent that were used in Figure 5B are shown within their respective chromosomal context. The FISH probes (red and green) cover the flanking regions of a DNA target region and are physically separated when a DNA double-strand break occurs (split signal). Images represent UCSC browser images.
